# Pregnancy-specific glycoproteins as potential drug targets for female lung adenocarcinoma patients

**DOI:** 10.1093/bfgp/elaf004

**Published:** 2025-04-21

**Authors:** Jung Hun Oh, Gabrielle Rizzuto, Rena Elkin, Corey Weistuch, Larry Norton, Gabriela Dveksler, Joseph O Deasy

**Affiliations:** Department of Medical Physics, Memorial Sloan Kettering Cancer Center, 321 E 61st Street, New York, NY 10065, United States; Human Oncology and Pathogenesis Program, Department of Pathology and Laboratory Medicine, Memorial Sloan Kettering Cancer Center, 1275 York Ave, New York, NY 10065, United States; Department of Medical Physics, Memorial Sloan Kettering Cancer Center, 321 E 61st Street, New York, NY 10065, United States; Department of Medical Physics, Memorial Sloan Kettering Cancer Center, 321 E 61st Street, New York, NY 10065, United States; Department of Medicine, Memorial Sloan Kettering Cancer Center, 1275 York Ave, New York, NY 10065, United States; Department of Pathology, Uniformed Services University of Health Sciences, 4301 Jones Bridge Rd, Bethesda, MD 20814, United States; Department of Medical Physics, Memorial Sloan Kettering Cancer Center, 321 E 61st Street, New York, NY 10065, United States

**Keywords:** pregnancy-specific glycoproteins, lung cancer, KRAS signaling pathway, differential sex effect

## Abstract

Recently, the mRNA presence of pregnancy-specific glycoproteins (PSGs) in cancer biopsies has been shown to be associated with poor survival. Given the pregnancy-related function of PSGs, we hypothesized that PSGs might act in a sex-dependent behavior in cancer patients. A differential sex effect of PSG genes with respect to tumor immune landscape and cancer outcomes was investigated using statistical, bioinformatic, and machine learning analyses in The Cancer Genome Atlas (TCGA) data. The resulting findings were then validated in the Clinical Proteomic Tumor Analysis Consortium (CPTAC) data. In a pan-cancer TCGA data analysis, the strongest PSG-related sex difference for the prognostic association was found in lung adenocarcinoma (LUAD). Kaplan–Meier analysis revealed that expression of PSG genes is strongly associated with overall survival rate in the female group on the TCGA, but not in the male group. This sex-specific association was validated in an independent dataset from the CPTAC study. A combination of PSG3, PSG7, and PSG8 expression was most significantly linked to poor prognosis in females (*P* = 8.67E-06 in TCGA and *P* = .0382 in CPTAC). Pathway analysis revealed enrichment of the ‘KRAS Signaling Down’ pathway in the high-risk female group. A predictive model showed good predictive performance for the female group (validated C-index = 0.78 in CPTAC), but poor predictive performance for the male group. These findings suggest that PSGs may have a sex-specific negative impact on survival in female LUAD patients, and the mechanism may be related to KRAS signaling pathway modulation.

## Introduction

Pregnancy-specific glycoprotein (PSG) genes are part of the carcinoembryonic antigen gene family which belongs to the immunoglobulin gene superfamily [1]. In humans, 10 PSG genes (PSG1–PSG9 and PSG11) located on chromosome 19q 13.1 to 13.3 encode closely related glycoproteins that are responsible for a broad range of immunoregulatory, pro-angiogenic, and anti-platelet functions in pregnant women [2, 3]. PSGs are secreted from syncytiotrophoblast cells which form the outer layer of the placenta and some extravillous trophoblasts, with increased concentrations as pregnancy progresses [4]. PSGs regulate innate and adaptive immunity and activate the latent form of TGF-β1 and TGF-β2 by binding to the corresponding latency-associated peptides [5–7]. However, little is known regarding PSG expression in non-pregnant individuals and its potential immunoregulatory activity [8].

Recently, some studies have reported that the PSG genes may play important roles in anti-tumor immunity and treatment response [9, 10]. Using a novel analysis method to model the whole genome-wide dependency of gene expression in The Cancer Genome Atlas (TCGA), Mathews *et al.* identified a network module with commonly expressed genes that includes 10 PSG genes and some other genes known to be expressed in the placenta. They subsequently found that PSG expression is likely associated with survival outcomes in lung adenocarcinoma (LUAD), breast, uterine, and colon cancers [9]. In a follow-up analysis, Zhao *et al.* showed significant survival differences in LUAD, breast, ovarian, and mesothelioma cancers between the high and low-risk groups defined by the ratio of expression levels of the PSG genes between the tumor and normal tissue from the Genotype-Tissue Expression [10]. They also found significant associations between the PSG genes and tumor immune cell abundance quantified on gene expression profiles, using CIBERSORT [11]. However, it can be questioned whether these relationships indicate true drivers of cancer mortality or just biomarkers of highly mutated and aggressive cancers.

In the current study, we hypothesized that there may be a differential sex effect of PSG genes with respect to tumor immune landscape and cancer outcomes. We first investigated the sex-dependent effect of PSGs in a pan-cancer TCGA analysis. We then conducted an in-depth study of the strongest sex-dependent effect seen in LUAD, using statistical, machine learning, and network-based analyses. We investigated whether PSGs are related to other known risk factors in lung cancer, using bioinformatics analysis. Finally, we defined a polygenetic risk signature that captures the sex-dependent PSG effect and validated the finding in the Clinical Proteomic Tumor Analysis Consortium (CPTAC) data [12].

## Methods

### RNA-Seq expression data

RNA-Seq expression profiles and relevant clinical data in the TCGA LUAD (N = 515) were downloaded from the UCSC Xena database (https://xenabrowser.net/datapages/) [13, 14]. For further validation of the findings in the TCGA LUAD, an independent dataset of 110 LUAD patients in the CPTAC study was analyzed using RNA-Seq expression profiles and relevant clinical data that were downloaded from the Genomic Data Commons (GDC) database (https://portal.gdc.cancer.gov/) [15]. Both RNA-Seq expression data were obtained from biospecimens collected before any treatment.

### Definition of PSG+/− status

For individual PSG genes, PSG+ was defined as ‘expression’ with its expression value >0 whereas PSG− was defined as ‘no expression’ with its expression value of 0*.*

### CIBERSORT analysis

CIBERSORT is a support vector regression-based deconvolution method that quantifies 22 immune cell types from RNA-Seq expression data [11]. CIBERSORT data for the TCGA LUAD were downloaded from the GDC database (https://gdc.cancer.gov/about-data/publications/panimmune) whereas CIBERSORT data for the CPTAC LUAD were computed using the R package immunedeconv (version 2.1.0) on RNA-Seq expression data [16].

### Bioinformatic analysis

Differential gene expression analysis was conducted to identify significantly different genes between the PSG+ and PSG− groups. Gene ontology analysis was then performed using the identified significantly different genes with the MSigDB hallmark gene set, consisting of 50 pathways [14]. GSEA was further conducted to compute enrichment scores for the resulting pathways.

### Wasserstein distance-based network analysis

Network analysis in connection with the Wasserstein distance was conducted to quantify inter-sample gene expression differences on a protein–protein interaction (PPI) network derived from the STRING database [17]. The Wasserstein distance is less sensitive to noise and outliers compared to traditional distance measures [18]. We used the *W*_1_-Wasserstein distance (also known as Earth Mover's distance: EMD), defined as follows:

${W}_1\left(\mu, \nu \right)=\underset{u}{\min}\left\{\sum_{i=1}^m\left\Vert{u}_i\right\Vert\ \left|\ \mu -\nu - Du=0\right.\right\}$ ,,where $\mu$and $\nu$ are two probability measures, $m$ is the number of edges in the network, ${u}_i$ are fluxes on the edges, and $D$ is an incidence matrix with rows and columns indexed by the nodes and edges in the network in which every entry $\left(i,j\right)$ is set to 1 if the node $i$ is assigned to be the head of the edge $j$ and is set to −1 if it is the tail of $j$ [19].

### Machine learning-based modeling

Machine learning-based modeling was performed utilizing penalized elastic net Cox regression and yielding C-index values with respect to overall survival [20]. This was performed using 10-fold cross-validation to find the optimal alpha value along with all default parameters in the CoxNetSurvivalAnalysis function from the sksurv Python package.

### Statistical analysis

Statistical analysis between the male and female groups was performed, using chi-squared and Wilcoxon rank-sum tests for categorical and continuous variables, respectively. Spearman correlation analysis was employed to assess the correlation between the PSG genes and CIBERSORT scores. Kaplan–Meier analysis with log-rank test was performed to evaluate the difference in overall survival between the PSG+ and PSG− groups for each sex. Univariate and multivariate Cox proportional hazards regression analyses were conducted to identify potential significant predictors for survival outcomes. In particular, multivariate Cox regression analysis was conducted using features with *P* < .05 in univariate analysis to reduce the risk of including irrelevant or weak predictors and minimize multicollinearity which can distort the model, while accounting for inter-feature relationships [21]. The Stata software was utilized for Cox proportional hazards regression analyses and Python packages were utilized for all other analyses in the Google Colab.

## Results

### Clinical characteristics

For the TCGA LUAD cohort, nine patients whose follow-up time was not available were excluded, resulting in 506, consisting of 235 male and 271 female patients, with median age of 66 years in both groups (*P* = .9844). There were no significantly different clinical variables between the male and female groups. More detailed clinical characteristics are shown in Table 1. For the CPTAC LUAD cohort, **four** patients whose follow-up time was not available were excluded, resulting in 106, consisting of 70 male and 36 female patients, with median age of 66 and 61 years (*P* = .2853), respectively.

**Table 1:** Clinical characteristics and their statistical tests between the male and female groups in the TCGA LUAD. Chi-squared and Wilcoxon rank-sum tests were employed for categorical and continuous variables, respectively. Each PSG gene was categorized into two groups with (+)/without (−) gene expression. IQR: Interquartile.

Variable	Status	Male (N = 235)	Female (N = 271)	*p*-value
Median age (IQR) (Years)		66 (59–72)	66 (59–73)	0.9844
Race	Black or African American	23	29	0.1227
	White	173	215	
	Other races	4	5	
	NA	35	22	
Stage	I	113	158	0.1159
	II	66	54	
	III	37	44	
	IV	14	12	
	NA	5	3	
Survival status at last follow-up	Alive	148	175	0.7093
	Dead	87	96	
Median follow-up (IQR) (Months)		20.5 (10.5–35.4)	21.6 (14.7–37.5)	0.2471
PSG1	Expression (+)	47	61	0.4921
	No expression (−)	188	210	
PSG2	Expression (+)	15	14	0.5570
	No expression (−)	220	257	
PSG3	Expression (+)	66	69	0.5057
	No expression (−)	169	202	
PSG4	Expression (+)	60	69	1.0000
	No expression (−)	175	202	
PSG5	Expression (+)	46	47	0.5179
	No expression (−)	189	224	
PSG6	Expression (+)	36	38	0.6801
	No expression (−)	199	233	
PSG7	Expression (+)	11	9	0.4337
	No expression (−)	224	262	
PSG8	Expression (+)	28	40	0.3493
	No expression (−)	207	231	
PSG9	Expression (+)	50	59	0.8933
	No expression (−)	185	212	
PSG11	Expression (+)	10	11	0.9128
	No expression (−)	225	260	

### Expression of PSG genes

**Figure 1:**
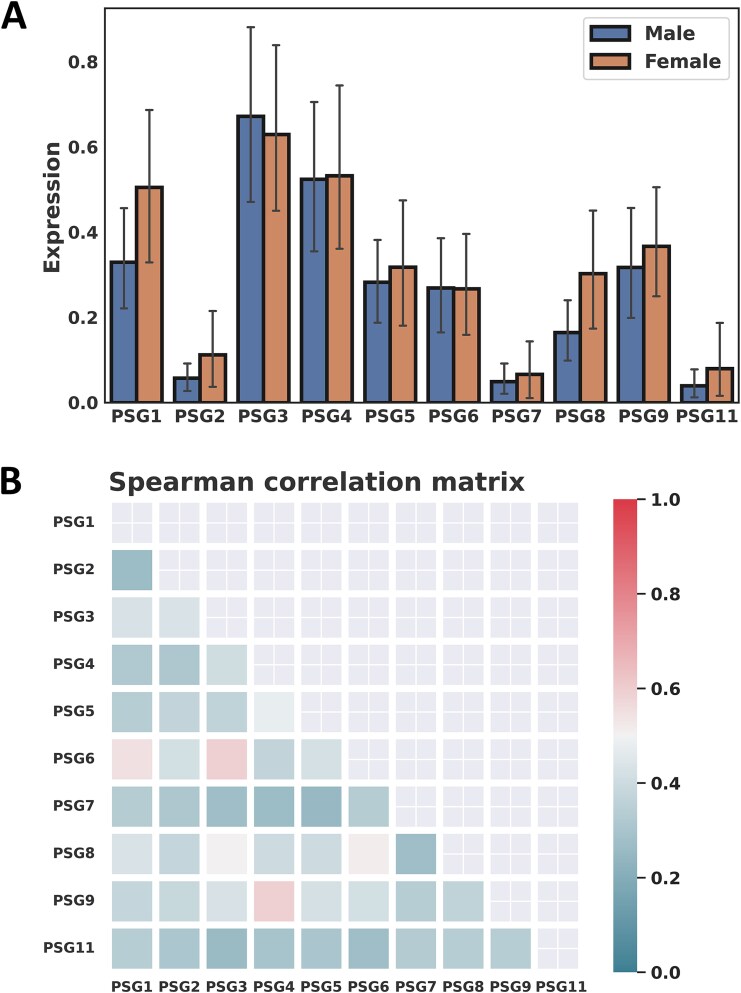
**Comparison of PSG genes. A** Comparison of expressions of the PSG genes between the male and female groups in the TCGA LUAD, which shows non-significant differences. **B** Heatmap of Spearman correlation coefficients between the PSG genes in the TCGA LUAD.

The PSG+/− status between the male and female groups in the TCGA LUAD was assessed and no significant difference was found for all the PSG genes (Table 1). In addition, there were no significant differences in expression of the PSG genes between the male and female groups (Fig. 1(a)). The PSG3 gene had the highest expression level in both male and female tumor samples whereas PSG2, PSG7, and PSG11 had relatively lower expression levels. Expressions of the PSG genes were compared between patients >50 and ≤ 50 years at age of diagnosis in each sex. In both sexes, there was a tendency that the younger group had increased expression compared to the older group (Supplemental Fig. 1). In particular, PSG2 and PSG9 had statistical significance with Wilcoxon rank-sum *P* = .0025 and .0307, respectively, in the female group. However, no statistically significant PSG gene was found in the male group.

Spearman correlation analysis was conducted between the PSG genes on the entire cohort in the TCGA LUAD, resulting in statistically significant *p*-values for all correlations. The statistical significance remained in all correlations of Spearman correlation analysis for each sex. The highest Spearman correlation coefficient (Rs) was observed between the PSG3 and PSG6 (Rs = 0.60, *P* < .0001) and the second highest Rs between the PSG4 and PSG9 (Rs = 0.59, *P* < .0001) as shown in Fig. 1(b). In Spearman correlation analysis between the PSG genes and 22 immune cell types in CIBERSORT, M0 macrophages yielded the four highest Rs in correlation with PSG5, PSG4, PSG9, and PSG6, resulting in Rs = 0.24, 0.23, 0.23, and 0.21 (all *P* < .0001), respectively (Supplemental Fig. 2).

### Survival analysis

**Figure 2:**
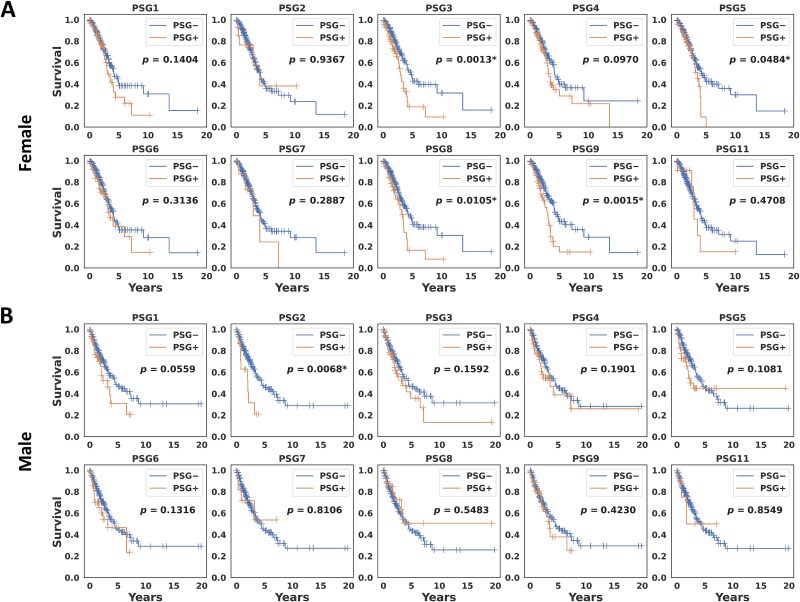
**Kaplan–Meier analysis of overall survival. A** The female and **B** male groups in the TCGA LUAD.

Kaplan–Meier analysis was conducted to investigate a difference in overall survival between the PSG+ and PSG− groups for each sex in the TCGA LUAD. Figure 2(a) shows Kaplan–Meier curves of overall survival with log-rank *p*-values for each PSG gene in the female group. Four genes, including PSG3, PSG5, PSG8, and PSG9, had statistical significance (*P* < .05) whereas, in the male group, only PSG2 had statistical significance but with a highly imbalanced sample size between the PSG+ (N = 15) and PSG− (N = 223) groups (Fig. 2(b)).

**Figure 3:**
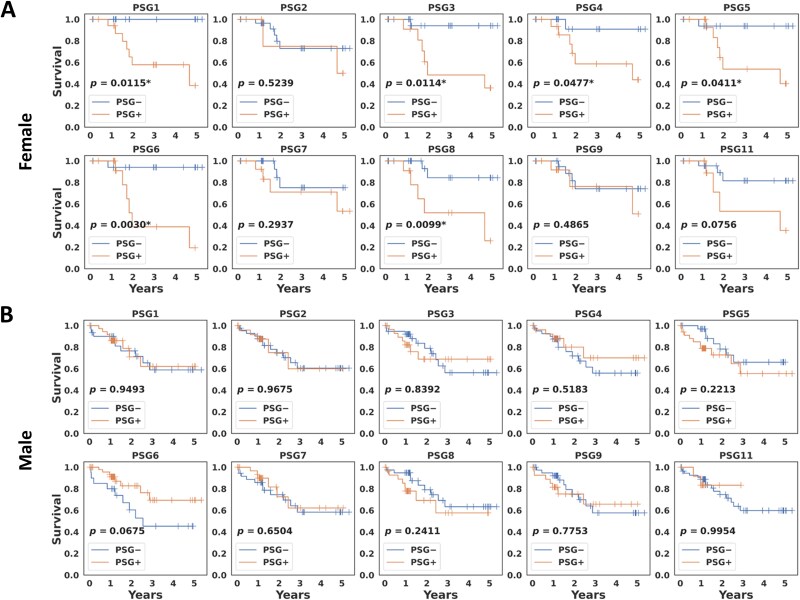
**Kaplan–Meier analysis of overall survival. A** The female and **B** male groups in the CPTAC LUAD.

An independent dataset in the CPTAC LUAD study was analyzed to validate the finding. Figure 3(a) shows Kaplan–Meier curves of overall survival with log-rank *p*-values for each PSG gene in the female group. Six PSG genes, including PSG1, PSG3, PSG4, PSG5, PSG6, and PSG8, had statistical significance whereas no statistically significant PSG gene between the PSG+ and PSG− groups was found in the male group (Fig. 3(b)). This finding suggests that there may be a differential sex effect of PSG genes on survival outcomes in LUAD. In particular, the observation that, in general, the PSG− group had higher survival probabilities than the PSG+ group indicates that expression of the PSG genes is associated with poor prognosis.

### Effect of the combination of PSG genes

**Figure 4:**
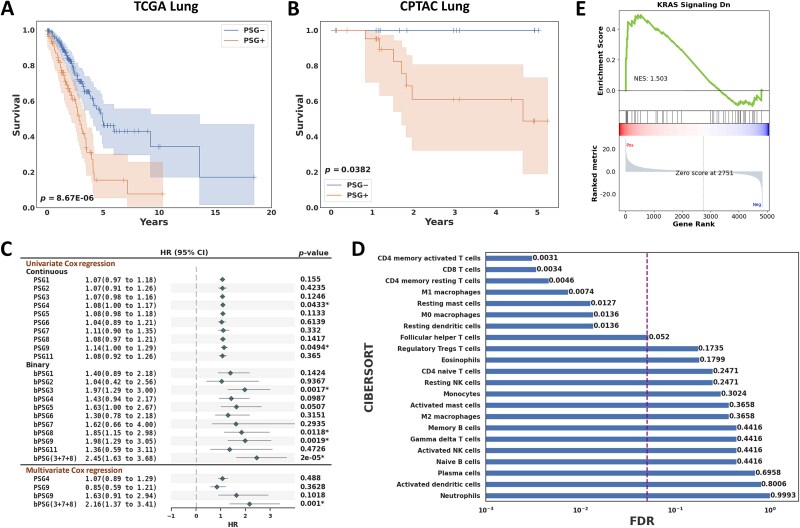
**Survival and bioinformatic analyses.** Kaplan–Meier analysis of overall survival for the female group when a tumor sample in which all PSG3, PSG7, and PSG8 genes had no expression was defined as PSG− and otherwise defined as PSG+ in the **A** TCGA LUAD and **B** CPTAC LUAD. **C** Univariate and multivariate Cox proportional hazards regression analyses on two feature sets of PSG data in the TCGA female LUAD: (**a**) the original gene expression values (continuous) and (**b**) binarized data as ‘no expression’ or ‘expression’ (coded as 0 and 1, respectively) as indicated with a prefix ‘b’. In the binarized feature set, an additional feature denoted as ‘bPSG (3 + 7 + 8)’ was added by coding a tumor sample in which all PSG3, PSG7, and PSG8 genes had no expression as 0 and otherwise 1. This predictor had the best univariate and multivariate performance. HR: Hazard ratio; CI: Confidence interval. **D** CIBERSORT analysis between the PSG+ and PSG− groups in the TCGA female LUAD. The vertical line indicates FDR = 0.05. **E** Gene set enrichment analysis (GSEA) of the ‘KRAS Signaling Down’ pathway identified in differential gene expression analysis between the PSG+ and PSG− groups in the TCGA female LUAD. A normalized enrichment score (NES) of 1.503 was obtained, indicating that the pathway was enriched by upregulated genes in the PSG+ group.

As mentioned earlier, expression of PSG genes is highly correlated, suggesting coordinated regulation in tumor cells and perhaps also coordinated mechanism of action. Indeed, PSGs could act in concert to regulate the tumor microenvironment. We next investigated whether certain combinations of PSG genes are associated with overall survival in Kaplan–Meier analysis. All possible combinations of two or three PSG genes (45 and 120 combinations, respectively) were assessed by defining a tumor sample as PSG− (70.1%, 190/271) if all PSG genes in a combination had no expression and otherwise defining it as PSG+ (29.9%, 81/271). In the female group on the TCGA LUAD, the lowest log-rank *p* of 8.67E-06 (false discovery rate [FDR] = 0.0010) was obtained with the combination of three PSG genes including PSG3, PSG7, and PSG8, which is much more statistically significant than individual PSG genes (Fig. 4(a)). This combination of PSG genes was validated on the CPTAC LUAD data, resulting in a log-rank *p* of 0.0382 (Fig. 4(b)). In the male group on the TCGA, no statistically significant combination with FDR < 0.05 was observed. The signature of PSG3, PSG7, and PSG8 was further assessed for female patients in other TCGA cancer datasets. A statistically significant survival difference was found between the PSG+ and PSG− groups in uterine cancer with a log-rank *p* of 0.0138 and borderline significance in kidney cancer with a log-rank *p* of 0.0746 (Supplemental Fig. 3).

### Cox proportional hazards regression analysis

To examine the impact of PSGs as a potential biomarker, Cox proportional hazards regression analysis was performed on two feature sets of PSG data in the TCGA LUAD: (a) the original gene expression values (continuous) and (b) binarized data as ‘no expression’ or ‘expression’ (coded as 0 and 1, respectively) as indicated with a prefix ‘b’. In the binarized feature set, an additional feature denoted as ‘bPSG (3 + 7 + 8)’ was added by coding a tumor sample in which all PSG3, PSG7, and PSG8 genes had no expression as 0 and otherwise 1. In the female group, univariate Cox regression analysis yielded marginal statistical significance for PSG4 (hazard ratio [HR] = 1.08, *P* = .0433) and PSG9 (HR = 1.14, *P* = .0494) on the original gene expression values (Fig. 4(C)). Interestingly, increased statistical significance was observed on the binarized features including bPSG3 (HR = 1.97, *P* = .0017), bPSG8 (HR = 1.85, *P* = .0118), bPSG9 (HR = 1.98, *P* = .0019), and bPSG (3 + 7 + 8) (HR = 2.45, *P* = 2E-05). Multivariate Cox regression analysis was performed on statistically significant features identified in univariate Cox regression analysis but excluding bPSG3 and bPSG8 because bPSG (3 + 7 + 8) likely includes redundant information from bPSG3 and bPSG8; bPSG (3 + 7 + 8) was highly correlated with bPSG3 and bPSG8, with Rs = 0.90 (*P* < .0001) and Rs = 0.64 (*P* < .0001), respectively. In multivariate Cox regression analysis using PSG4, PSG9, bPSG9, and bPSG (3 + 7 + 8), only bPSG (3 + 7 + 8) had statistical significance with *P* = .0010 (HR = 2.16). In the male group, univariate Cox regression analysis yielded statistical significance for PSG2 (HR = 2.39, *P* = .0023), bPSG1 (HR = 1.66, *P* = .0495), and bPSG2 (HR = 2.58, *P* = .0078). However, no statistically significant feature was found in multivariate Cox regression analysis with the three features (Supplemental Table 1).

### CIBERSORT analysis

To explore the possible relationship between PSGs and tumor immune landscape, CIBERSORT analysis was performed by assessing differences in estimated tumor immune cell abundance between the PSG+ and PSG− groups in TCGA female LUAD patients (grouped by the signature of PSG3, PSG7, and PSG8 as shown in Fig. 4(a)). Note that a tumor sample in which PSG3, PSG7, and PSG8 had no expression was defined as PSG− and otherwise was defined as PSG+. Of the 22 immune cell types in CIBERSORT, seven obtained statistical significance including CD4 memory activated T cells (FDR = 0.0031), CD8 T cells (FDR = 0.0034), M1 macrophages (FDR = 0.0074), and M0 macrophages (FDR = 0.0136) with higher abundance in the PSG+ group and CD4 memory resting T cells (FDR = 0.0046), resting mast cells (FDR = 0.0127), and resting dendritic cells (FDR = 0.0136) with higher abundance in the PSG− group (Fig. 4(D)).

### Biological pathway analysis

To broadly investigate whether PSGs were related to the change of biological pathways, differential gene expression analysis between the PSG+ and PSG− groups in TCGA female LUAD patients (grouped by the signature of PSG3, PSG7, and PSG8 as shown in Fig. 4(a)) was performed on RNA-Seq expression profiles downloaded from the UCSC Xena database [13]. With a log2 fold change >2 and an FDR < 0.05, 266 genes were identified (Supplemental Table 2). Gene ontology analysis was then performed using the Molecular Signatures Database (MSigDB) hallmark gene set [22]. Of the total 50 pathways analyzed, only the ‘KRAS Signaling Down’ pathway, consisting of the gene set downregulated upon KRAS activation, showed statistical significance (*P* = .0008, FDR = 0.0178). Gene set enrichment analysis (GSEA) was further conducted on the pathway, using a set of genes with FDR < 0.05, which yielded a normalized enrichment score (NES) of 1.503, indicating that the pathway was enriched by upregulated genes in the PSG+ group (Fig. 4(e)). We then asked if this pattern (a statistically significant alteration of the ‘KRAS Signaling Down’ pathway between the PSG+ and PSG− groups in female LUAD patients grouped by the signature of PSG3, PSG7, and PSG8) is present in other TCGA cancer datasets. Interestingly, thyroid cancer alone was found to have a similar statistical significance for the ‘KRAS Signaling Down’ pathway (*P* < .0001, FDR < 0.0001) in the PSG+ versus PSG− groups.

### Network analysis using the Wasserstein distance

**Figure 5:**
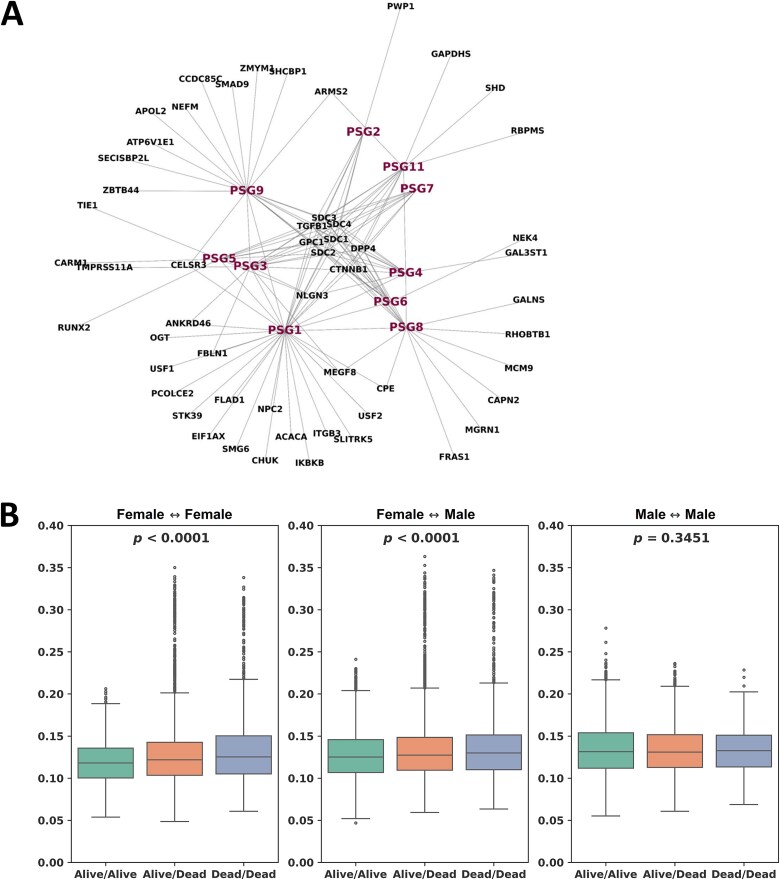
**Network analysis. A** Protein–protein interaction network that consists of the 10 PSG genes and their 54 interacting genes. **B** Wasserstein distance computed for pairs of patients on the protein–protein interaction network in the TCGA LUAD. Gene expression pattern variability in the neighborhood of the PSGs was greater for female LUAD patients.

To characterize the biological similarity of genes known to have close interactions with the PSGs, we adopted a network analysis approach. Modeling was conducted on a PPI network that consists of the 10 PSG genes and their 54 interacting genes derived from the STRING database [17]. Figure 5(a) illustrates the resulting PPI network. Prior to the analysis, patients with follow-up time less than the median follow-up time (1.8 years) were excluded, which left 254 patients with 114 male (78 alive and 36 deceased) and 140 female patients (89 alive and 51 deceased) in the TCGA LUAD.

Wasserstein distance between each pair of patients was computed on the PPI network. The resulting distances between the female patients were categorized into three groups (alive/alive, alive/dead, and dead/dead) based on survival status at last follow-up time. Gene expressions of the PSG neighborhood were more similar in pairs of patients who were both alive compared to pairs with at least one deceased patient, yielding a statistically significant *P* < .0001 in the analysis of variance (ANOVA) test (Fig. 5(b)). A similar finding was observed between the female and male patients. In contrast, no statistical significance was found between the male patients (*P* = .3451). Hence, gene expression pattern variability in the neighborhood of the PSGs was greater for female patients.

### Predictive modeling

To evaluate predictive power of the identified biomarkers, machine learning-based modeling was conducted for male and female groups, separately, in the TCGA LUAD and the resulting models were tested in the independent CPTAC LUAD data. A model based on the presence of expression in any of the PSG genes 3, 7, or 8 was developed for the female group in the TCGA and applied to the CPTAC data, yielding a C-index of 0.70, whereas the resulting model for the male group yielded poor predictive performance (C-index = 0.56). For internal validation in the female group, the TCGA data were stratified into two sets: a training set (2/3 of samples) and a validation set (1/3 of samples), while keeping nearly the same survival rate and follow-up time distribution in each set. This resulted in a C-index of 0.65.

To test the relationship between PSGs and tumor immune response, principal component analysis (PCA) was conducted on 22 immune cell types in CIBERSORT, and a model was developed for the female group in the TCGA, using three principal components (PCs) derived from CIBERSORT together with PSG genes and their related genes, which provided little improvement (CPTAC validation C-index = 0.71) in predictive performance. The ‘KRAS Signaling Down’ pathway was identified as a potential mechanism in female LUAD patients using a gene ontology analysis with respect to the expression in any of the PSG genes 3, 7, or 8. To clarify the relationship between the PSG biomarker and ‘KRAS Signaling Down’ pathway, PCA was conducted on gene expressions for a set of genes associated with the pathway. A model developed using the biomarker candidate (indicating the presence of expression in any of the PSG genes 3, 7, or 8) and three PCs of the pathway improved the CPTAC validation C-index to 0.78 in the female group, with poor predictive performance (C-index = 0.59) in the male group.

## Discussion

In the current study, we demonstrated the sex-dependent effect of PSGs on cancer survival outcomes based on a pre-planned hypothesis informed by previously reported findings. PSGs are secreted by trophoblast cells and are believed to play critical immunomodulatory and anti-inflammatory roles to prevent rejection of the fetus, with increased concentrations as pregnancy progresses [23]. Several studies have examined PSGs to better understand the underlying mechanisms of maternal immune tolerance to the fetus [24–26]. Wojtowicz et al. identified several potential PSG binding partners including PDGFRA, FGFR4, CLEC4A, DCC, and DSCAM although the implication of these associations and whether they truly exist *in vivo* have not been investigated [27]. Several studies have reported that PSGs activate the latent form of the anti-inflammatory cytokine transforming growth factor (TGF)-β1, potentially contributing to the conversion of naïve CD4^+^ T cells into regulatory T cells (Tregs) to maintain immune tolerance [23, 28, 29]. The ability to increase the percentage and number of CD4^+^ Tregs was confirmed for two PSGs, PSG1 and PSG9, although it is likely to be shared by the others [23, 28–30]. Snyder et al. showed that PSGs induce the secretion of IL-10 and TGF-β1 by human monocytes [31]. PSGs have also been shown to impact non-immune functions during pregnancy as some PSGs were reported to bind to α5β1 integrin, increasing trophoblast adhesion and migration, and to have pro-angiogenic activity by interacting with heparan sulfate proteoglycans (including syndecans) on endothelial cells [7]. In our additional analysis, no significant differential sex effect on LUAD survival was found for sexually dimorphic and hormone-related genes, despite reports of sex-specific cancer biomarkers in other studies. Li et al. found significant differences in mutation density and the frequency of mutations in specific cancer driver genes between male and female tumors [32]. Shin et al. also reported sex-specific cancer biomarkers in several cancers, suggesting strategies for sex-biased therapy [33].

PSGs were reported to have relatively higher expression levels in a majority of tumors compared to normally very low levels in normal tissues [10]. Previous studies have shown that elevated expression of PSGs in tumor specimens is associated with poor survival [9, 10]. Given both the pregnancy-related action of PSGs on the maternal immune system and the several documented sex differences in immunity [34], we hypothesized that the PSG impact on survival could be sex-dependent. We first asked whether there are any sex-related differences in the association between PSGs and survival across multiple cancers. Strong associations between PSG expression and survival were found for female patients in LUAD, uterine, and kidney cancers, with the largest effect seen in LUAD. We therefore conducted further analyses in LUAD. To test whether this was a causal relationship, predictive modeling with respect to overall survival was conducted, and the resulting model was validated in an independent dataset. As reported above, a model based on the presence of expression in any of the PSG genes 3, 7, or 8 resulted in promising predictive performance (C-index = 0.70) for female LUAD patients, and poor predictive performance (C-index = 0.56) for male LUAD patients.

We also tested the hypothesis that PSGs affect host immune cells present within tumors. To examine this, we constructed a predictive model based on immune-related variables derived from CIBERSORT analysis together with PSG genes and their interacting genes. Although there is a clear pattern relating PSGs to immune variables, as reported elsewhere [10], the resulting model provided little improvement (C-index = 0.71) for the female group. We therefore expanded our search for potential mechanisms in female LUAD patients by looking into all known pathways and their relationships with PSGs. After a gene ontology analysis, only the ‘KRAS Signaling Down’ pathway showed a statistically significant difference between the PSG+ and PSG− groups shown in Fig. 4(a). To further clarify the relationship between PSGs as risk factors and the ‘KRAS Signaling Down’ pathway, we examined whether the biomarker candidate (indicating the presence of expression in any of the PSG genes 3, 7, or 8) and three PCs of the pathway could better predict overall survival. The resulting model for the female group improved the predictive performance to C-index = 0.78.

Expression of individual PSG genes has been shown to be associated with poor survival. In Kaplan–Meier analysis on the TCGA LUAD, survival rates for the PSG genes 3, 5, 8, and 9 were significantly different between the female PSG+ (‘expression’) and PSG− (‘no expression’) groups, whereas, in the male group, only PSG2 had statistical significance but with a highly imbalanced sample size between the PSG+ (N = 15) and PSG− (N = 223) groups. These findings were validated on the CPTAC LUAD data; in the female group, the same analysis yielded six statistically significant PSG genes 1, 3, 4, 5, 6, and 8, resulting in three common statistically significant PSG genes (PSG3, PSG5, PSG8) in the TCGA and CPTAC, with higher survival probabilities in the PSG− group. However, no statistically significant PSG gene was found in the CPTAC male cohort. Note that there was no significant difference in expression of the PSG genes between the male and female groups, implying that the sex-dependent effect is independent of PSG expression levels between male and female patients (Fig. 1(a)). This suggests that while there is no evidence for sex-associated ligands of PSGs, the biological mechanisms of PSGs, particularly their interactions with the KRAS pathway and tumor immune system, may differ between male and female patients, with better prognostic benefit in female patients with lower expression of the PSG genes. Recently, a few studies have reported potential sex-specific targets which are likely to be associated with a sex bias in treatment response. Richtmann et al. showed that higher pregnancy-associated protein glycodelin (encoded by the PAEP gene) levels in tumors correlate with significantly worse survival outcomes in female non-small-cell lung cancer (NSCLC) patients treated with immunotherapy, but this was not observed in male patients, suggesting that glycodelin could be used as a promising immunological biomarker to early identify female NSCLC patients who do not benefit from the immunotherapy [35]. Ahrenfeldt et al. reported that the increased adaptive-to-innate immune ratio is associated with improved overall survival in both male and female patients through a pan-cancer analysis, but with a stronger association in female patients, suggesting a sex bias in treatment response [36].

Strong correlations among the PSG genes observed in Fig. 1(b) may imply that they closely interact with each other to regulate tumor immune tolerance. However, not much is known about how PSG expression might affect cancer-related immunity [9]. In analysis of the combinatorial effect of PSG genes, a combination of PSG3, PSG7, and PSG8, denoted as ‘bPSG (3 + 7 + 8)’, yielded the lowest *p* of 8.67E-06 (FDR = 0.0010) between the female PSG+ and PSG− groups in the TCGA LUAD and was validated with *P* = .0382 in the CPTAC LUAD data. The signature of PSG3, PSG7, and PSG8 was also found to show a statistically significant survival difference (*P* = .0138) between the PSG+ and PSG− groups in uterine cancer and borderline significance (*P* = .0746) in female kidney cancer patients.

In CIBERSORT analysis between the female PSG+ and PSG− groups in the TCGA LUAD, seven immune cell types were statistically significantly different, including CD4 memory activated T cells, CD8 T cells, M1 macrophages, and M0 macrophages with higher abundance in the PSG+ group and CD4 memory resting T cells, resting mast cells, and resting dendritic cells with higher abundance in the PSG− group. Some of these associations, while previously reported [10], are unexpected as some PSGs have been shown to induce an alternative M2-like macrophage phenotype [6] and an increase in CD4^+^ T-regulatory cells *in vivo* and *in vitro* [37]. In gene ontology analysis, the ‘KRAS Signaling Down’ pathway was found to be statistically significant, showing its enrichment in the PSG+ group by upregulated genes. This finding was also observed in TCGA thyroid cancer. It has been reported that KRAS signaling shapes the immunosuppressive nature of the tumor microenvironment [38, 39]. Hu et al. reported that KRAS signaling drives immune evasion by activating CD47 in LUAD [40].

Taken together, our results lend support to the novel hypothesis that PSGs exert sex-specific effects on tumor-immune cell crosstalk and cancer outcomes, suggesting that PSGs could be drug targets for ~30% of female LUAD patients with PSG expression. However, the current study has some limitations that need to be discussed. Defining PSG+ and PSG− based on zero expression is a simple approach that allows for straightforward analysis. Identifying an optimal cut-point is desirable and a subject of future research. More studies should be performed to explore sex-specific therapeutic targets and treatment strategies and to investigate the potential of our identified biomarkers for other cancer outcomes and early diagnosis. The current study is limited to mRNA expression of the PSG genes and should be validated with analysis of PSG expression with anti-PSG antibodies. Exon 2 in PSG7 contains rs113247044 (G/A) with a major allele (A) frequency of 83% and nearly 70% of individuals have an AA genotype. The A allele results in a stop codon which causes early termination of translation. Furthermore, it has been reported that the non-coding PSG7 transcript may act as a lncRNA, similar to PSG10P; however, this has not been tested [7, 41]. In addition, the biological interplay of PSGs and KRAS signaling pathway as well as their involvement in the tumor immune modulation should be further examined through wet-lab experiments.

**Key Points**

- Expression of PSGs has a sex-specific negative impact on survival in female LUAD patients.
- A combination of PSG3, PSG7, and PSG8 expression was most significantly linked to poor prognosis in females.
- The mechanism is related to KRAS signaling pathway modulation.

## Supplementary Material

Supplemental_Figures_elaf004

Supplemental_Table1_elaf004

Supplemental_Table2_elaf004

## Data Availability

RNA-Seq expression profiles for the TCGA LUAD were downloaded from the UCSC Xena database (https://xenabrowser.net/datapages/). RNA-Seq expression profiles for the CPTAC LUAD were downloaded from the GDC database (https://portal.gdc.cancer.gov/). CIBERSORT score data for the TCGA LUAD were downloaded from the GDC database (https://gdc.cancer.gov/about-data/publications/panimmune).
