# Supplementary material for: Pregnancy-specific glycoproteins as potential drug targets for female lung adenocarcinoma patients
Source: Brief Funct Genomics. 2025 Apr 21;24:elaf004. doi: 10.1093/bfgp/elaf004 (PMC12010166; doi:10.1093/bfgp/elaf004)
Supplement: Supplemental_Figures_elaf004 [file supplemental_figures_elaf004.docx]

**
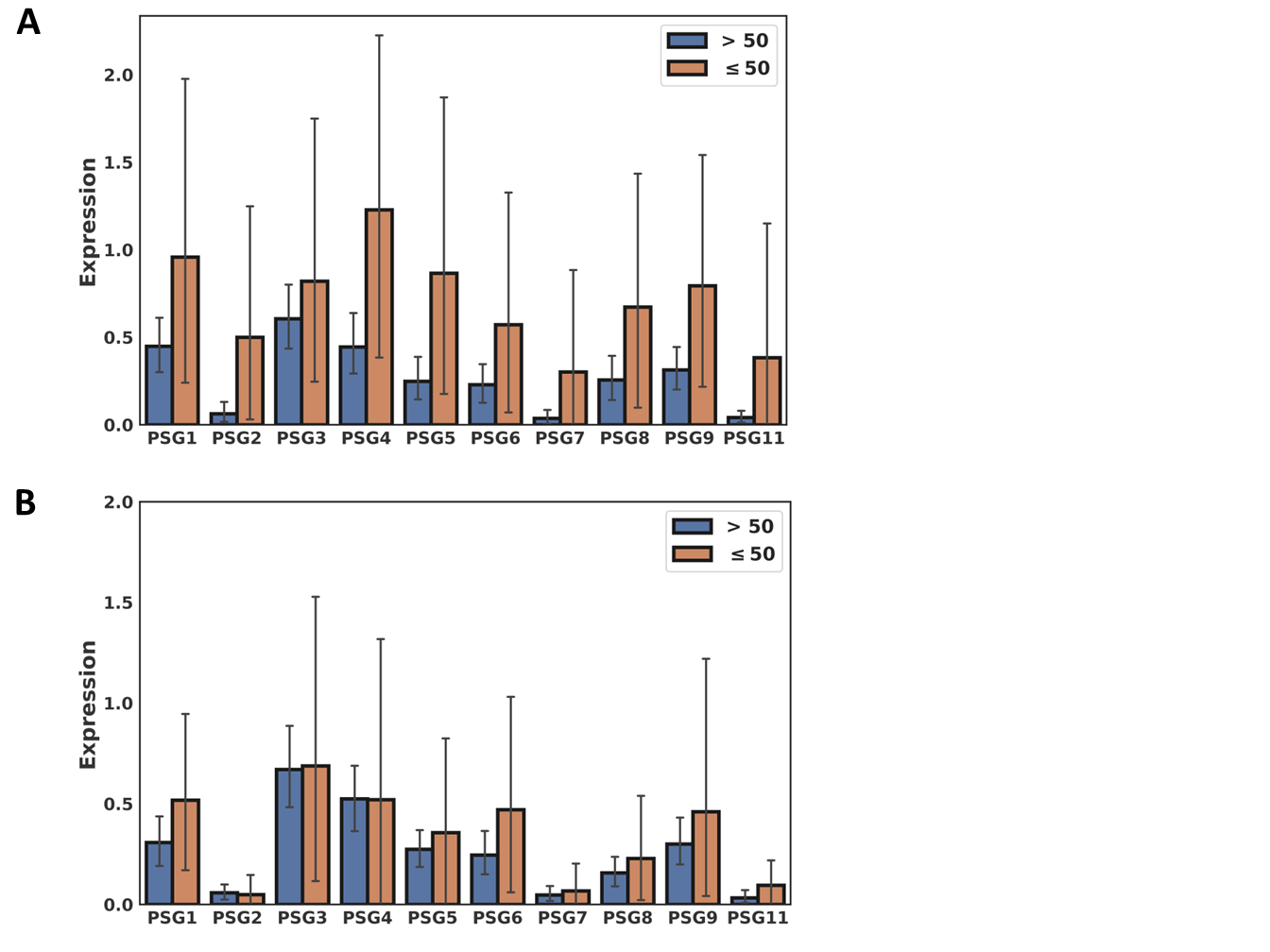
**

**Figure 1.** Comparison of expressions of the PSG genes between patients > 50 and ≤ 50 years at age of diagnosis for **A** the female and **B** male groups in the TCGA LUAD.


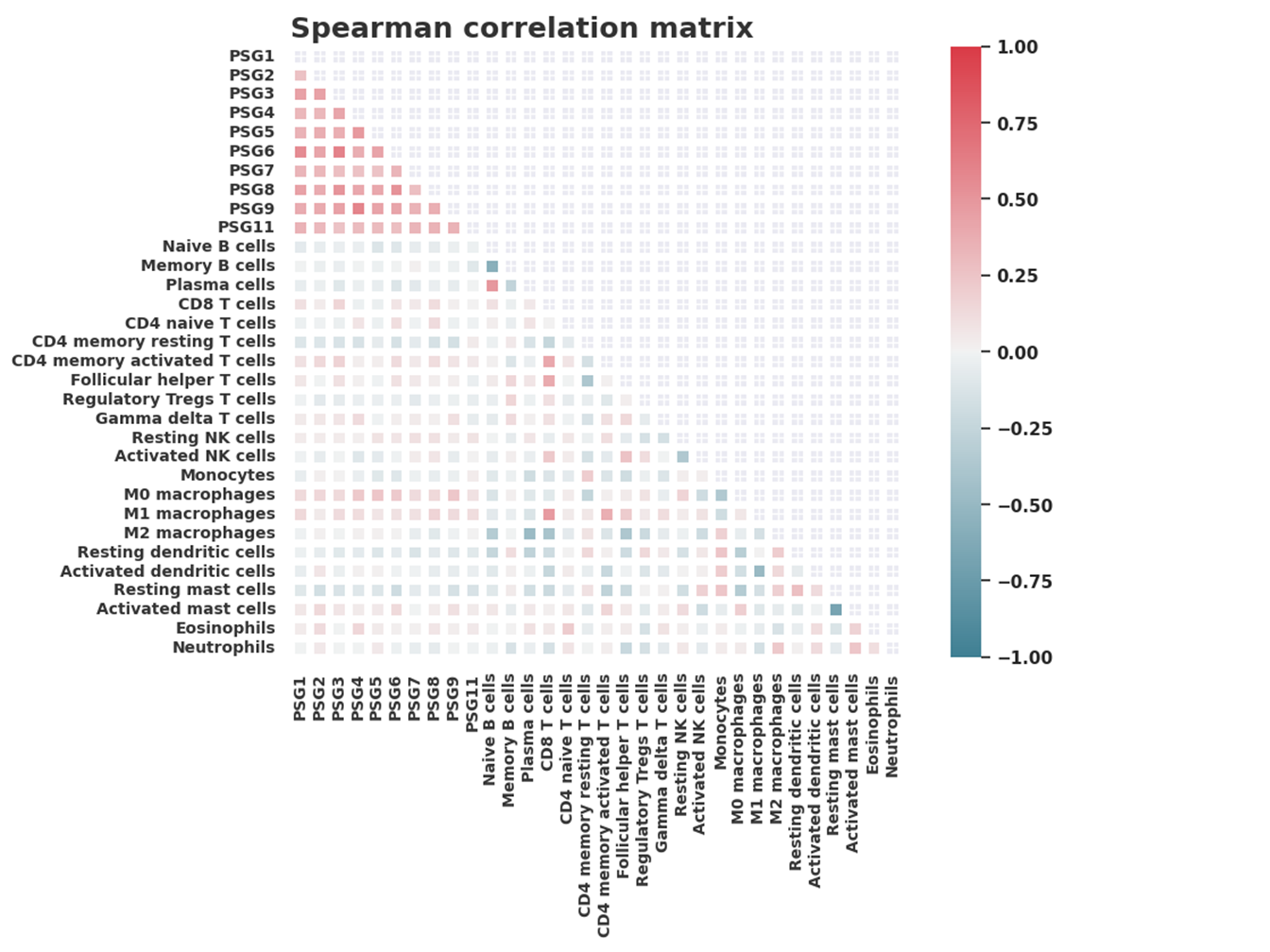


**Figure 2.** A heatmap of Spearman correlation coefficients between the PSG genes and CIBERSORT scores in the TCGA LUAD.


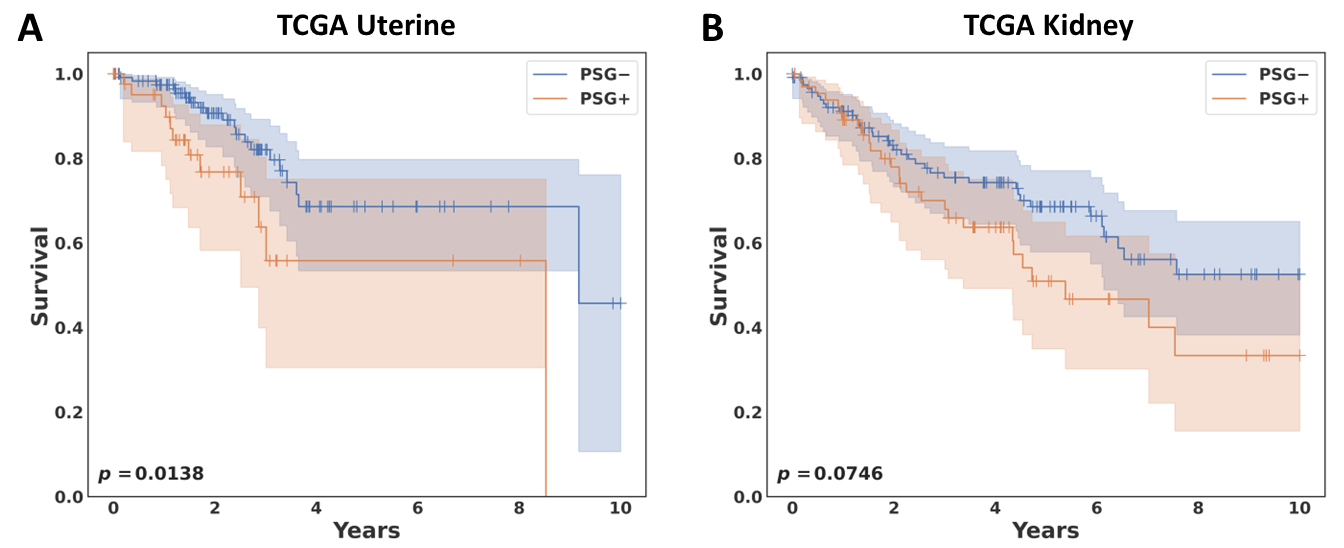
 **Figure 3.** Kaplan-Meier analysis of overall survival for the female group when a tumor sample in which all PSG3, PSG7, and PSG8 genes had no expression was defined as PSG− and otherwise defined as PSG+ in the **A** TCGA uterine cancer and **B** TCGA kidney cancer.
